# Australia’s War against Rabbits: The Story of Rabbit Haemorrhagic Disease

**DOI:** 10.3201/eid2104.142009

**Published:** 2015-04

**Authors:** Paul Effler

**Affiliations:** Associate Editor, Emerging Infectious Diseases; University of Western Australia, Perth, Western Australia, Australia

**Keywords:** Australia, New Zealand, parasites, rabbit hemorrhagic disease, viruses

In Australia’s War against Rabbits, Brian Cooke details the emergence of rabbit hemorrhagic disease (RHD) in Asia and Europe and subsequent efforts to introduce RHD virus into Australia and New Zealand for rabbit control in the 1990s. The book documents the ecologic adaption between a virus and its animal host and provides cogent examples of our inability to contain the spread of (what was thought to be) a well-understood virus after it escaped into the environment.

The first failed containment effort ensued after the virus jumped from a quarantine compound on Wardang Island to the coast of South Australia 3 km away. Despite a program carefully designed to counter unplanned spread of RHD virus on the Australian mainland (Operation Garter), the virus prevailed. Uncontained spread also followed an intentional and illegal release of RHD virus in New Zealand in 1997 when a farmer with intractable rabbit problems allegedly smuggled the virus in from Australia. After the outbreak started, instead of helping the New Zealand government control RHD, farmers reportedly used blenders to make “rabbit smoothies” and actively spread RHD virus by applying these slurries to carrot baits.

The book is generally well written and heavily referenced and contains many anecdotes penned by a scientist obviously passionate about his work. The flow of the narrative can sometimes be erratic, however, veering from amusing personal stories to a didactic recounting of the taxonomy of rabbit parasites or legislation relevant to pest control in Australia. Australia’s War against Rabbits is best suited for professionals with a keen interest in rabbits, fleas, and their pathogens.

